# Speed of heart rate changes during postural provocations in children and adolescents

**DOI:** 10.1038/s41598-024-62000-7

**Published:** 2024-05-24

**Authors:** Martina Šišáková, Kateřina Helánová, Katerina Hnatkova, Irena Andršová, Tomáš Novotný, Marek Malik

**Affiliations:** 1 Department of Internal Medicine and Cardiology, University Hospital Brno, Jihlavská 20, 625 00 Brno, Czech Republic; 2 Department of Internal Medicine and Cardiology, Faculty of Medicine, Masaryk University, Jihlavská 20, 625 00 Brno, Czech Republic; 3 National Heart and Lung Institute, Imperial College, 72 Du Cane Rd, Shepherd’s Bush, London, W12 0NN England

**Keywords:** Computational biology and bioinformatics, Developmental biology, Physiology, Cardiology

## Abstract

Heart rate is under constant autonomic influence but the development of the influence in children is not fully understood. Continuous electrocardiograms were obtained in 1045 healthy school-age children (550 females) during postural provocations with body position changes between supine, sitting, standing, supine, standing, sitting and supine (in this order), 10 min in each position with position changes within 20 s. Heart rate was measured in each position and speed of heart rate changes between positions were assessed by regressions of rates versus timing of individual cardiac cycles. Supine heart rate was gradually decreasing with age: 82.32 ± 9.92, 74.33 ± 9.79, 67.43 ± 9.45 beats per minute (bpm) in tertile age groups < 11, 11–15, > 15 years, respectively (*p* < 0.0001), with no significant sex difference. Averaged speed of heart rate changes differed little between sexes and age groups but was significantly faster during rate deceleration than acceleration (e.g., supine ↔ standing: 2.99 ± 1.02 vs. 2.57 ± 0.68 bpm/s, *p* < 0.0001). The study suggests that in children, vagal heart rate control does not noticeably change between ages of approximately 6–19 years. The gradual resting heart rate decrease during childhood and adolescence is likely caused by lowering of cardiac sympathetic influence from sympathetic overdrive in small children to adult-like sympatho-vagal balance in older adolescents.

## Introduction

As well known, heart rate is under constant autonomic control^1^. While sympathetic drive increases heart rate, in addition to the effects on myocardial contractility, bronchodilatation, and blood vessel tone, parasympathetic effects decrease heart rate through the direct influence of the left-sided vagus nerve on the sinus node^2^. While basal heart rate free of autonomic influence is around 110–120 beats per minute (bpm)^1^ vagal effects decrease the spontaneous frequency of sinus node depolarisation and reduce the heart rate to the usual 50–70 bpm^3^. The tone of efferent sympathetic fibres has the opposite effect and accelerates heart rate by increasing the sinus node depolarisation rate^1,4^.

It is also well known that resting heart rate in normal children decreases with age from approximately 120–130 bpm at birth to close to the normal adult rate levels around late puberty^5^. This has been explained by gradual development and maturation of the parasympathetic system and reflexes in children. It has been reported that parasympathetic activity increases from infancy to middle (girls) or late (boys) childhood, followed by a plateau phase and with subsequent slight decrease in adolescence^6^. The pattern of sympathetic activity maturation is much less known and linear decrease with increasing age has been described^6^.

While fully plausible, the reported evidence of gradual development of the parasympathetic influence on the sinus node is indirect^6,7^. Other possibilities might be proposed including age-related changes of the basal heart rate, increased resting sympathetic tone in young children, and oversensitivity of the developing sinus node to normal beta-adrenergic tone.

To contribute to the understanding of the development of the autonomic influence on heart rate during childhood, we have hypothesised that the level of autonomic responsiveness of sinus nodal periodicity would be optimally investigated during autonomically active provocative manoeuvres that lead, in fully developed hearts, to rapid heart rate changes. Specifically, we proposed that if the vagal and sympathetic systems are not fully mature in young children, their heart rate responses to autonomic provocations would not only lead to numerically smaller heart rate changes but that these heart rate changes would also occur at noticeably slower speeds.

For this purpose, we developed a technology for measuring the speed of heart rate changes in continuous electrocardiograms (ECG). We applied this technology to recordings obtained in a sufficiently large population of children and adolescents investigated during a series of strictly defined postural provocations.

## Methods

### Study population

The study investigated healthy children and adolescents of school age. The recruitment aimed at obtaining uniform age distribution between the ages of 4–19 years in both sexes. The participants were enrolled into the study at preparatory, primary, and secondary schools in southern Silesia and Moravia (countries within the Czech Republic) offering ECG-based health checks. While every child or adolescent who agreed to participate was investigated, the data used in the analysis presented here excluded those who were on pharmaceuticals potentially affecting cardiac electrophysiology or on hormonal contraceptives, those with cardiac abnormality, and participants undergoing sex transversal procedures. All subjects whose data were analysed in the study has normal physical investigation. The study protocol was approved by the Ethics Committee of the University Hospital Brno on 13th June 2018 without any numerical approval reference. Written informed consent was obtained from all participants who were legally allowed to provide it. For other participants, written informed consent was obtained from their parents or legal guardians. All aspects of the study adhered to the requirements of the Helsinki declaration. Standard demographic data were collected for all participants.

### Postural provocations and ECG recordings

Continuous 12-lead ECG (SEER MC version 2, sampled at 1000 Hz) with electrodes in the Mason-Likar position^8^ was recorded in each participant during 70-min provocative postural manoeuvres. These consisted of a series of postural changes between different body positions. These comprised supine, sitting, standing, supine, standing, sitting, and supine positions, each of 10-min duration, and were adopted in this order. The sitting and standing positions were maintained without any back support. Per protocol, the position changes were accomplished within no more than 20 s.

All participants were investigated in the mid-morning hours in groups of up to 20 subjects of similar ages performing the positional changes at the same time. During the investigation, younger children were listening to non-exciting age-appropriate fairy tales, others were investigated in quiet noise-free environment. During the protocol, the participants were not allowed to speak and non-verbal contacts within the investigated groups were supressed as much as possible.

For feasibility reasons, the ECG recordings were started before the provocative manoeuvring and terminated afterwards. While the duration of the ECG recordings before and after the provocative manoeuvring differed between subjects, the timing of the recordings was documented allowing to identify the different position phases in each continuous ECG.

During the morning hours prior to the recording, the participants followed standard education-related activities which did not include any physical exercise. None of the participants smoked before or during the ECG recording.

### QRS detection and heart rate measurements

To facilitate computer processing of the ECG signals, each of the continuous recordings was divided into 10-s segments with 5-s overlaps between neighbouring segments. In each segment, individual QRS complexes were identified in the digital recordings using 4 different detection algorithms that implemented previously proposed methods applied to both the native ECG signals and their derivatives^9–12^. When these detection algorithms provided mutually consistent results, the results were accepted for further analyses. If the different algorithms provided inconsistent results, the sequence of the QRS complex positions in the 10-s segment was reviewed visually and, where necessary, manually corrected. The same visual/manual review/correction was applied when inconsistencies were found in the overlapping sections of neighbouring segments. A previously reported, in-house developed and validated graphics software was used for review/correction^13^. The visual/manual review/correction was performed while the operators were blinded in respect of the timing of the reviewed 10-s segments and of the corresponding demographic data of the study subject.

Using the QRS positions in individual 10-s segments, a continuous series of QRS positions was obtained for each recording. For the measurement of heart rate in any sub-section of the recording, mean RR interval was calculated and converted into heart rate value expressed in bpm. Among others, subject-specific heart rate was measured in 8 middle minutes of each postural position (i.e., the first and the last minute of the 10-min postural position were excluded to eliminate instabilities). These heart rate values were interpreted as stable heart rates of each postural position.

### Speed of heart rate changes

**Figure 1:**
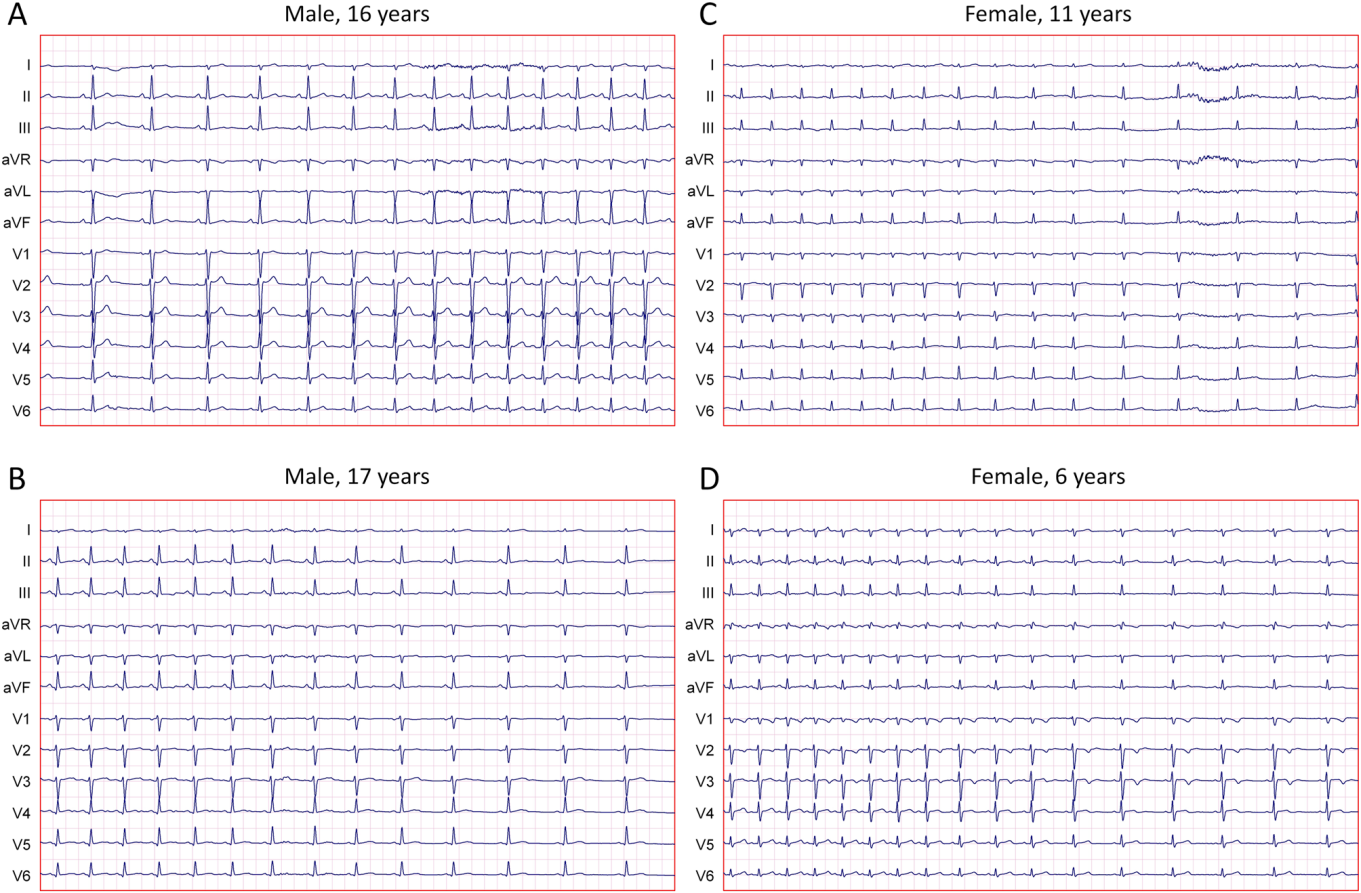
Examples of significant heart rate acceleration/deceleration. Examples of linear slopes of heart rate values related to the RR interval positions. Cases (**A**) and (**B**) show examples of narrow slope confidence intervals, cases (**C**) and (**D**) show examples with wider slope confidence intervals which still showed slopes statistically significantly different from 0. Cases (**A**) (male, aged 17 years), (**B**) (male, 16 years), (**C**) (female, 11 years) and (**D**) (female, 6 years) showed slopes − 6.87 bpm/s (with 95% confidence interval − 6.93 to − 5.74 bpm/s), + 6.83 (+ 6.27 to + 7.40) bpm/s, − 9.38 (− 10.87 to − 7.90) bpm/s, and − 9.58 (− 11.35 to − 7.83) bpm/s, respectively.

The speed of heart rate changes was assessed in 10-s segments of the continuous ECG recording (i.e., in any 10-s segment, not only in segments used to detect the QRS complex positions). For this purpose, linear regression analysis between the RR interval positions and durations was used while the RR interval durations were expressed as heart rate values in bpm. That is, if a 10-s segment of the ECG recording contained QRS positions at time moments ${\left\{{t}_{i}\right\}}_{i=0}^{n}$ (measured in seconds), slope of linear regression was obtained between heart rate values ${\left\{60/({t}_{i+1}-{t}_{i})\right\}}_{i=0}^{n-1}$ and centres of the individual RR intervals ${\left\{({t}_{i+1}+{t}_{i})/2\right\}}_{i=0}^{n-1}$. The slopes of the linear regressions were evaluated together with their confidence intervals (examples in Fig. 1). The confidence intervals of the slopes allowed to distinguish between ECG segments that showed true heart rate accelerations or decelerations and segments showing only RR interval irregularity.

Based on these slopes, a QRS complex was considered followed by heart rate acceleration if the 10-s interval starting with the corresponding QRS position showed positive heart rate slope with positive lower 80% confidence interval of the slope. Correspondingly, a QRS complex was considered followed by heart rate deceleration if the 10-s interval starting with the corresponding QRS position showed negative heart rate slope with negative upper 80% confidence interval of the slope.

Using this definition, the following characteristics of heart rate change were derived in each subject for each postural position change.

#### Maximum heart rate slope

For each QRS position withing ± 1 min of the moment of the per-protocol postural change, maximum heart rate acceleration slope (for position changes supine → sitting, sitting → standing, and supine → standing) or minimum heart rate deceleration slope (for position changes standing → supine, standing → sitting, and sitting → supine) was found and expressed as the absolute bpm/s value.

#### Duration of heart rate change

Around the position of the QRS complex that showed the maximum absolute heart rate slope, a search was performed to find all consecutive QRS complexes that were all followed by heart rate acceleration/deceleration. The time difference between the first and last these QRS complexes defined the duration of systematic heart rate change.

#### Cumulative heart rate slope

A curve of absolute heart rate slopes versus time was constructed using the slopes of all consecutive QRS complexes that were all followed by heart rate acceleration/deceleration (as used in the definition of the heart rate change duration) with the slope values assigned to the time instances of the QRS complexes. The area under this curve was divided by the duration of the heart rate change to obtain the averaged heart rate slope. (That is, the cumulative heart rate slope was an average of slopes assigned to individual QRS complexes weighted by corresponding RR interval durations.)

#### Absolute heart rate change

Heart rate was calculated over 3-min intervals between 4 and 1 min before and between 1 and 4 min after the moment of the per-protocol postural change. Absolute value of the difference between these heart rates defined the absolute heart rate change of the postural manoeuvre.

### Statistics and data presentation

Continuous data are presented as mean ± standard deviation. All the analysed data show bell-shaped distributions (and thus the mean ± standard deviation presentation is appropriate) but do not necessarily satisfy Kolmogorov–Smirnov normality test in all cases.

The study population was divided into approximate tertiles according to the age of the subjects. In each of these age strata, heart rate and rate change characteristics were compared between sexes using non-parametric two-sample Mann–Whitney test. Separately for each sex, differences between the age tertiles were tested using Kruskal–Wallis one-way ANOVA test.

Intra-subject differences in characteristics were calculated between heart rate acceleration and deceleration measured in corresponding postural changes, i.e., between supine → sitting and sitting → supine, sitting → standing and standing → sitting, and between supine → standing and standing → supine. These intra-subject differences were evaluated using one-sample Wilcoxon signed-rank test, their relationship to age was tested using non-parametric Spearman’s correlation coefficients that were evaluated together with their 95% confidence intervals (CI) based on Fisher’s r-to-z transformation.

ECG data processing was performed by in-house developed software routines programmed in C/C++ (Microsoft Visual Studio 2022, version 17.4.3). Statistical evaluations were performed using IBM SPSS Statistics package (version 29.0.0.0). P-values were considered statistically significant if below 0.05. Because of the interdependency of the compared data, no significance adjustment for multiplicity of test was performed. All statistical tests performed are reported.

STROBE checklist^14^ was followed when composing the text of the article.

## Results

### Population and electrocardiographic data

The investigation according to the study protocol was performed in 1094 subjects. Of these, 49 (4.5%) were subsequently excluded for reasons that we have already described (see the Methods sub-section on Study population). The study population thus included 1045 subjects of whom 550 were females aged 12.92 ± 3.64 years and 495 were males aged 13.03 ± 3.56 years (no significant difference between the ages of female and male participants). Among the study population, 26 subjects (15 females and 11 males) did not complete the protocol investigation because of physical discomfort, pre-syncopal symptoms, or nausea with occasional vomiting in small children. The partial data of these subjects were used in the analyses presented here.

**Figure 2:**
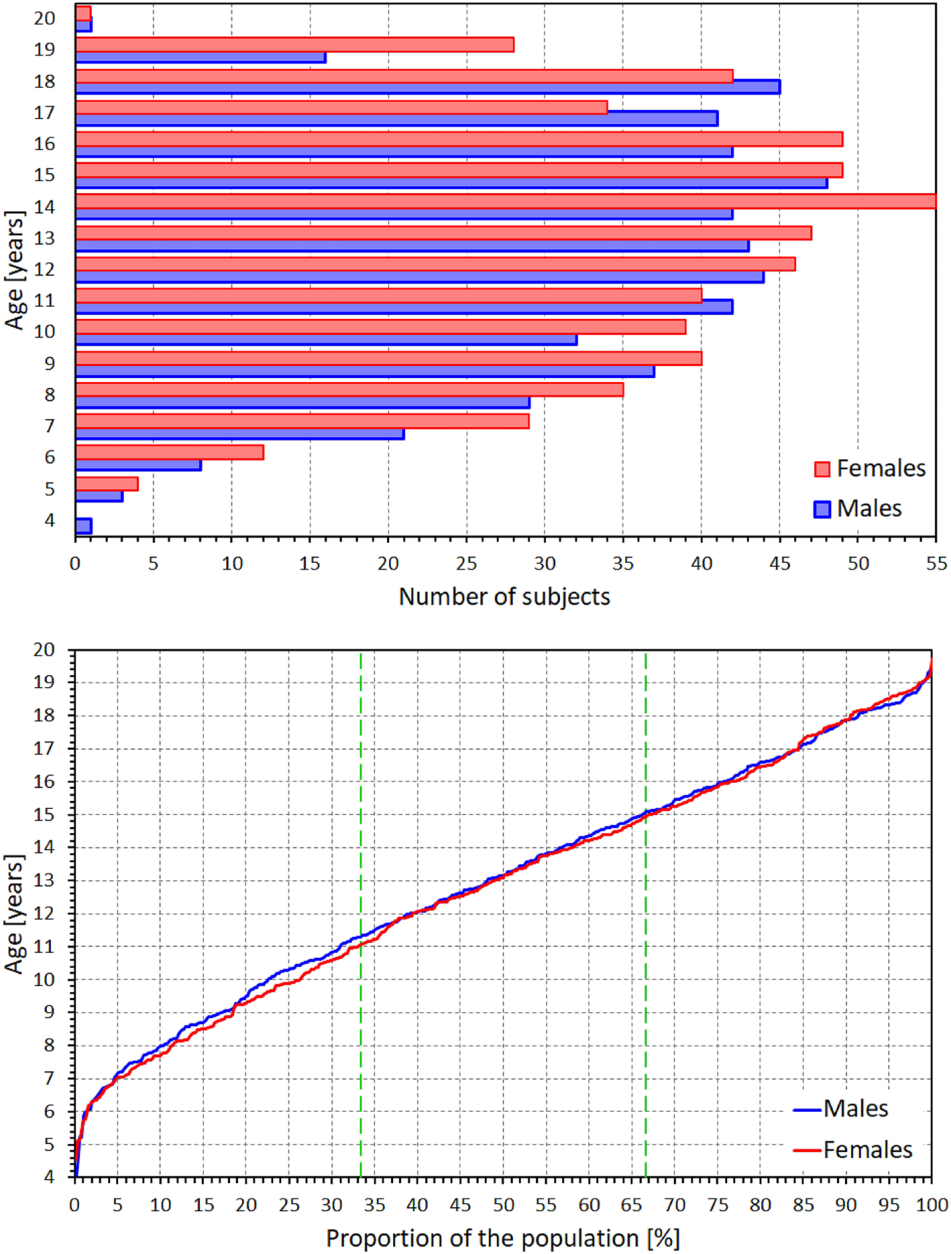
Composite of the study population. The top panel shows the number of subjects in different age groups (age of each subject rounded to the closest integer), the bottom panel shows cumulative distributions of the ages. Vertical green dashed lines in the bottom panel indicate the tertiles of the populations used in the study.

The age distribution of the study population is shown in Fig. 2. The bottom part of the figure shows that between ages of 6 and 19 years, the age distribution was practically uniform in both sexes.

Figure 2 also shows that the tertiles of the study population were reasonably defined using age dichotomies of below 11 years (181 females and 153 males), between 11 and 15 years (186 females and 174 males), and above 15 years (183 females and 168 males), respectively.

### Individual postural provocations

#### Heart rates

**Figure 3:**
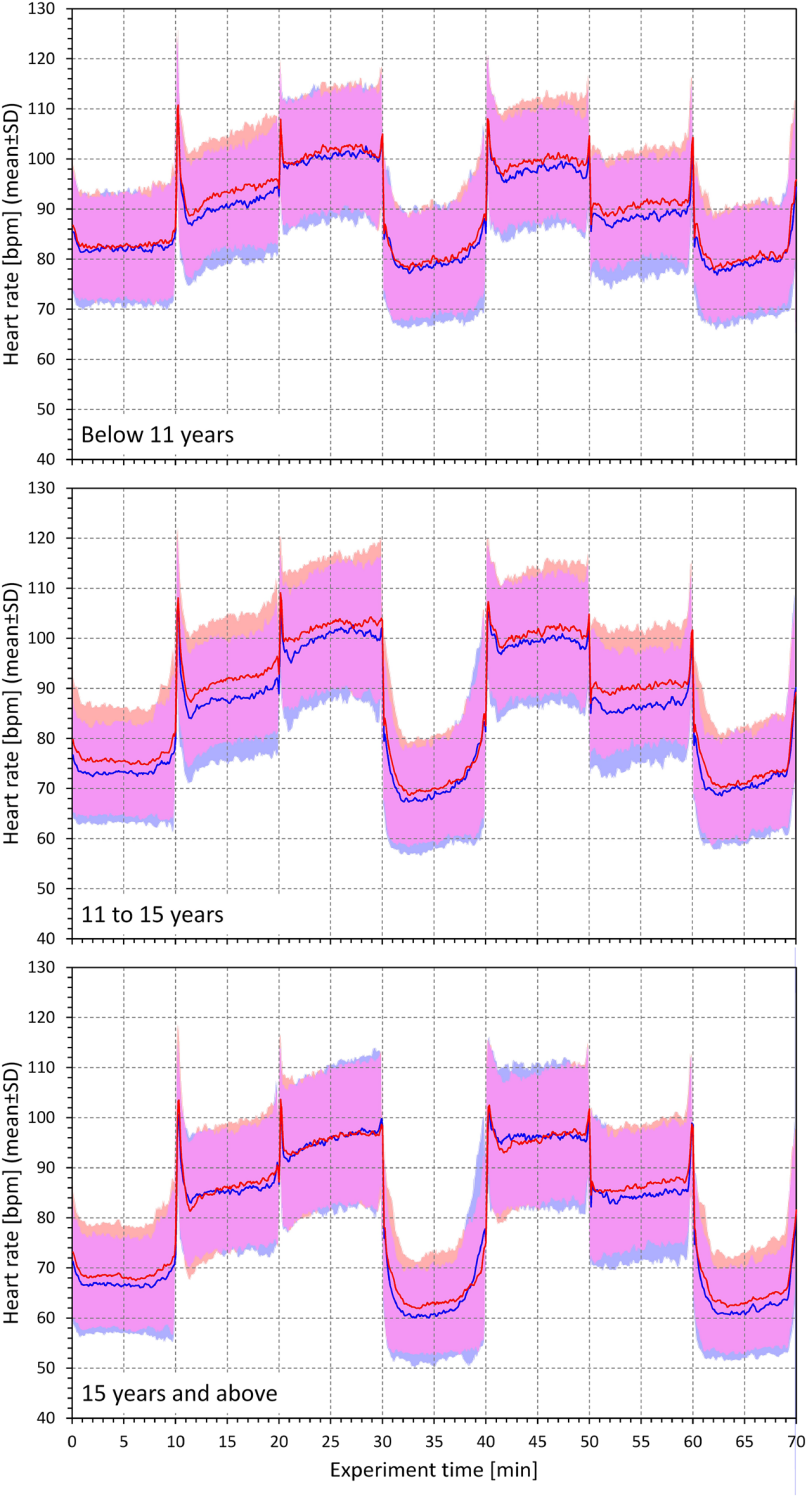
Heart rates measured during the study protocol. Heart rates in beats per minute (bpm) measured during the 7 phases of the study that corresponded to the following postural positions: supine → unsupported sitting → unsupported standing → supine → unsupported standing → unsupported sitting → supine. In each subject, the time axis was shifted to align the maximum heart rate changes in the complete population. The red and blue bold lines show mean values in females and males, respectively; the red and blue bands show mean ± standard deviation in females and males, respectively; the violet bands show the overlap between the mean ± standard deviation bands of both sexes. The top, middle, and bottom panels correspond to the age groups of below 11 years, 11–15 years, and above 15 years, respectively.

Stable heart rates of each postural position are shown in Fig. 3 and Table 1. Minimal heart rate differences with only occasional statistical significance were seen between sexes. On the contrary, the difference between the age-defined tertiles of the population was statistically significant at all positions.

**Table 1:** Stable heart rates in different postural positions.

	Female	Male	*P*-value (F vs. M)
Supine 1			
Age < 11 years	82.56 ± 9.72	82.04 ± 10.17	0.6435
Age 11–15 years	75.40 ± 10.20	73.18 ± 9.22	0.0628
Age > 15 years	68.27 ± 9.83	66.51 ± 8.95	0.0610
*P*-value (age groups)	< *0.0001*	< *0.0001*	
Sitting 1			
Age < 11 years	92.83 ± 10.30	90.44 ± 10.59	*0.0337*
Age 11–15 years	91.23 ± 11.43	87.62 ± 11.33	*0.0035*
Age > 15 years	85.86 ± 12.18	85.01 ± 11.39	0.2474
*P*-value (age groups)	< *0.0001*	< *0.0001*	
Standing 1			
Age < 11 years	101.60 ± 11.00	100.77 ± 11.75	0.5491
Age 11–15 years	102.45 ± 11.86	100.3 ± 11.95	0.1888
Age > 15 years	95.65 ± 12.78	95.65 ± 13.25	0.9834
*P*-value (age groups)	< *0.0001*	*0.0005*	
Supine 2			
Age < 11 years	79.58 ± 9.43	78.77 ± 10.00	0.4696
Age 11–15 years	70.48 ± 9.84	69.07 ± 9.65	0.3367
Age > 15 years	63.31 ± 9.37	61.12 ± 8.76	*0.0467*
*P*-value (age groups)	< *0.0001*	< *0.0001*	
Standing 2			
Age < 11 years	99.63 ± 10.86	97.95 ± 10.54	0.2639
Age 11–15 years	101.23 ± 11.56	99.59 ± 11.58	0.2275
Age > 15 years	95.80 ± 12.51	96.25 ± 13.22	0.5594
*P*-value (age groups)	*0.0001*	*0.0044*	
Sitting 2			
Age < 11 years	90.57 ± 9.54	88.17 ± 10.65	*0.0409*
Age 11–15 years	90.25 ± 10.63	86.16 ± 10.21	*0.0003*
Age > 15 years	86.48 ± 11.63	84.41 ± 12.41	*0.0399*
*P*-value (age groups)	*0.0006*	*0.0031*	
Supine 3			
Age < 11 years	79.85 ± 9.39	78.99 ± 9.51	0.6967
Age 11–15 years	71.82 ± 9.82	70.54 ± 9.18	0.0681
Age > 15 years	63.71 ± 9.42	61.78 ± 8.65	*0.0115*
*P*-value (age groups)	< *0.0001*	< *0.0001*	

Heart rate values shown (mean ± standard deviation) in beats per minute. *P*-values “F versus M” show statistical comparison of both sexes, “age groups” show inter-sex comparisons of age-tertiles. Statistically significant P-values are shown in italics.

The differences between age-defined tertiles were numerically largest at supine positions (more than 15 bpm) while fairly minimal (less than 5 bpm, albeit still statistically significant) during the standing positions—see Table 1.

#### Heart rate changes

**Figure 4:**
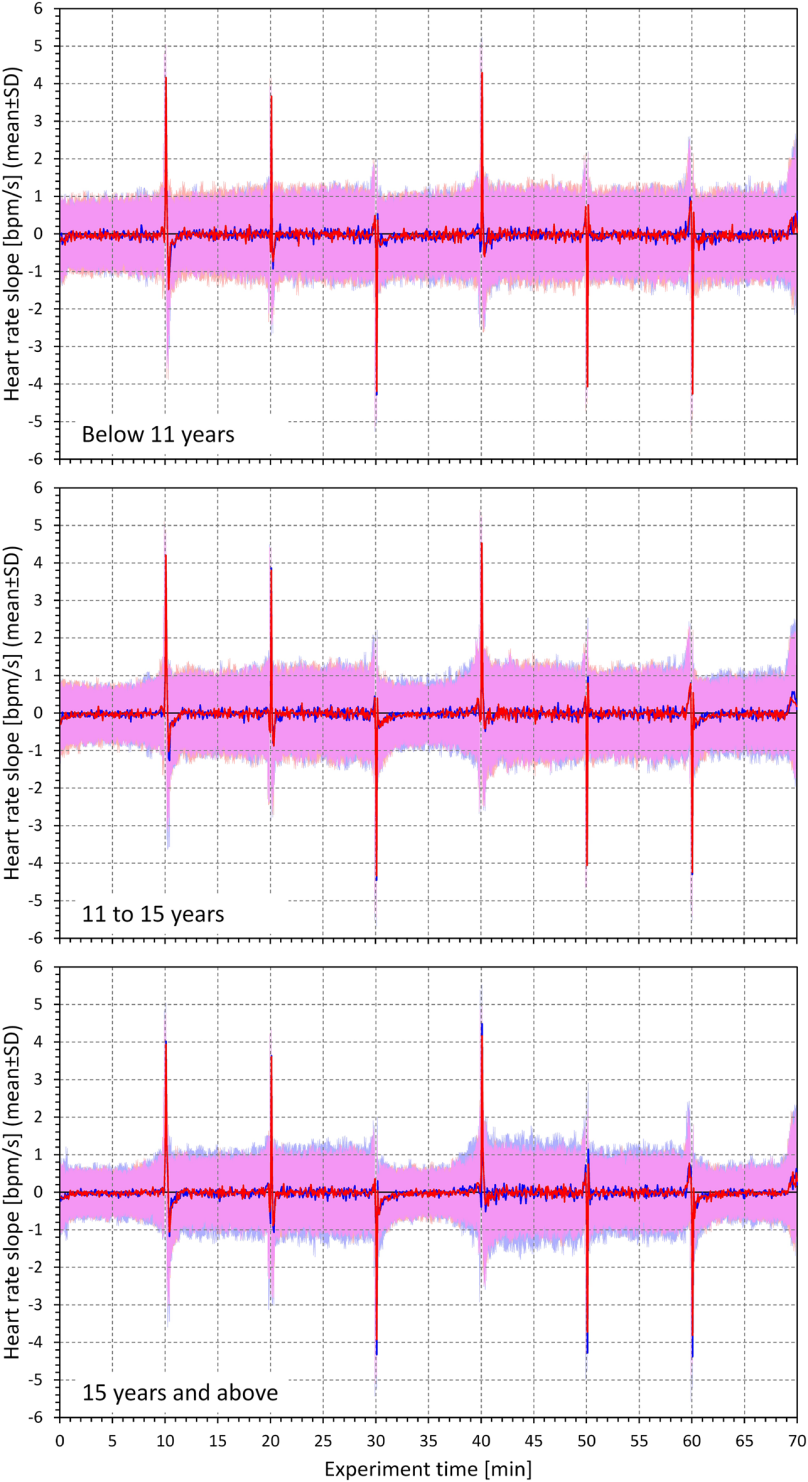
Heart rate changes measured during the study protocol. Heart rate changes in beats per minute (bpm) changes per second (i.e., expressed by heart rate/time slopes—see the main text for explanation) measured during the 7 positions phases of the study. The layout of the figure is the same as in Fig. 3.

Figure 4 shows the development of 10-s heart rate slopes during the study recordings. The comparison of Fig. 4 with Fig. 3 shows that while there were substantial heart rate differences in the age-defined tertiles of the population, little differences existed between the speed of heart rate changes.

The numerically small differences between the speed of heart rate changes are confirmed in Tables 2 and 3 which show the characteristics of heart rate changes during the postural transitions between the supine and standing positions. Corresponding data of the other postural transitions are shown in Supplementary tables 1 to 4. Contrary to the expectations, the speed of heart rate changes was not found systematically faster in older subjects in comparison to the young children. In older subjects there was a noticeable trend towards faster heart rate changes in males compared to females, but this trend has not always reached statistical significance.

**Table 2:** Profiles of heart rate deceleration during the standing → supine position change.

	Female	Male	p-value (F vs. M)
Maximum slope [bpm/s]			
Age < 11 years	4.27 ± 1.16	4.39 ± 1.26	0.3826
Age 11–15 years	4.56 ± 1.62	4.84 ± 2.43	0.4490
Age > 15 years	4.01 ± 1.39	4.43 ± 1.37	*0.0005*
*P*-value (age groups)	*0.0010*	0.5382	
Cumulative slope [bpm/s]			
Age < 11 years	2.97 ± 0.81	3.02 ± 0.88	0.6365
Age 11–15 years	3.06 ± 1.01	3.28 ± 1.38	0.1811
Age > 15 years	2.67 ± 0.9	2.96 ± 0.96	*0.0013*
*P*-value (age groups)	*0.0001*	0.1710	
Heart rate change duration [s]			
Age < 11 years	7.59 ± 3.42	7.44 ± 3.17	0.5936
Age 11–15 years	8.03 ± 3.92	8.22 ± 4.29	0.6733
Age > 15 years	9.03 ± 4.78	8.90 ± 4.67	0.8645
*P*-value (age groups)	*0.0438*	*0.0278*	
Rate change [bpm]			
Age < 11 years	23.66 ± 10.18	23.27 ± 8.81	0.8606
Age 11–15 years	33.53 ± 11.51	33.31 ± 11.14	0.7763
Age > 15 years	34.04 ± 10.46	36.41 ± 12.60	0.1342
*P*-value (age groups)	< *0.0001*	< *0.0001*	

Absolute values of heart rate change measurements shown as mean ± standard deviation. *P*-values “F versus M” show statistical comparison of both sexes, “age groups” show inter-sex comparisons of age-tertiles. bpm—beats per minute. Statistically significant *P*-values are shown in italics.

**Table 3:** Profiles of heart rate acceleration during the supine → standing position change.

	Female	Male	*P*-value (F vs. M)
Maximum slope [bpm/s]			
Age < 11 years	4.17 ± 0.90	4.17 ± 0.90	0.8612
Age 11–15 years	4.35 ± 1.02	4.41 ± 1.04	0.6652
Age > 15 years	4.02 ± 0.86	4.36 ± 0.97	*0.0003*
*P*-value (age groups)	*0.0053*	0.1950	
Cumulative slope [bpm/s]			
Age < 11 years	2.59 ± 0.63	2.54 ± 0.65	0.3429
Age 11–15 years	2.62 ± 0.79	2.68 ± 0.66	0.2302
Age > 15 years	2.39 ± 0.6	2.61 ± 0.72	*0.0076*
*P*-value (age groups)	*0.0018*	0.2315	
Heart rate change duration [s]			
Age < 11 years	10.53 ± 5.98	10.5 ± 5.69	0.7481
Age 11–15 years	12.44 ± 7.04	11.93 ± 7.17	0.3851
Age > 15 years	15.05 ± 7.87	13.41 ± 8.74	*0.0343*
*P*-value (age groups)	< *0.0001*	0.0612	
Rate change [bpm]			
Age < 11 years	18.35 ± 8.68	17.44 ± 7.71	0.7351
Age 11–15 years	28.12 ± 9.81	28.14 ± 10.21	0.7580
Age > 15 years	30.46 ± 10.67	34.09 ± 12.22	*0.0073*
*P*-value (age groups)	< *0.0001*	< *0.0001*	

Absolute values of heart rate change measurements shown as mean ± standard deviation. *P*-values “F versus M” show statistical comparison of both sexes, “age groups” show inter-sex comparisons of age-tertiles. bpm—beats per minute. Statistically significant *P*-values are shown in italics.

**Figure 5:**
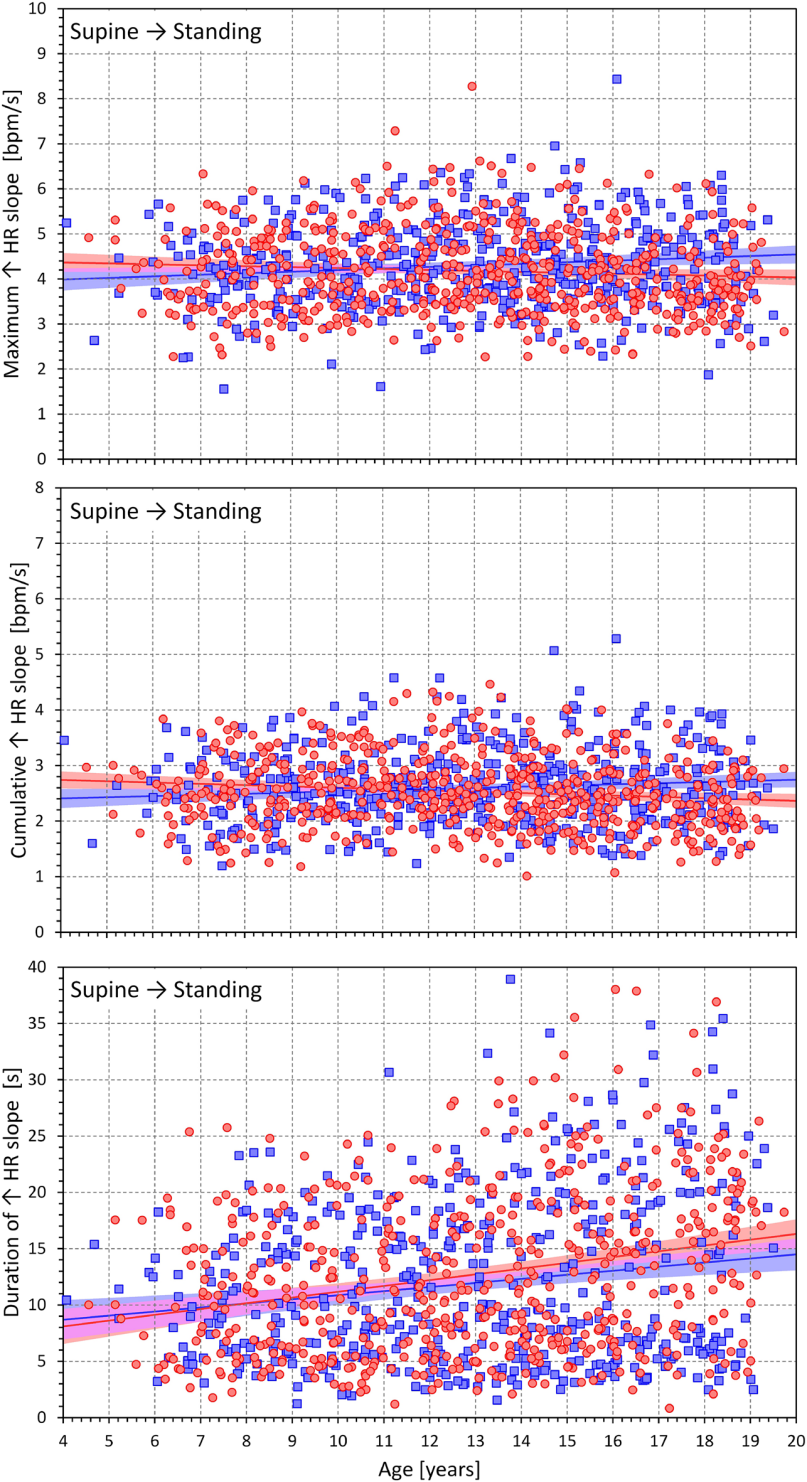
Dependency of heart rate acceleration slopes on the age of study participants. Maximum heart rate slopes (top panel), cumulative heart rate slopes (middle panel), and the duration of heart rate slopes (bottom panel) measured during the position change from supine to standing related to the age of study subjects. In each panel, the red circles and blue squares correspond to female and male participants, respectively; the red and blue bold lines are linear regression between the displayed values and the age of the female and male subjects, respectively; the light red and light blue bands are 95% confidence intervals of the linear regressions in female and male subjects, respectively. The violet bands are the overlaps between the confidence intervals of both sexes. HR—heart rate; bpm—beats per minute.

**Figure 6:**
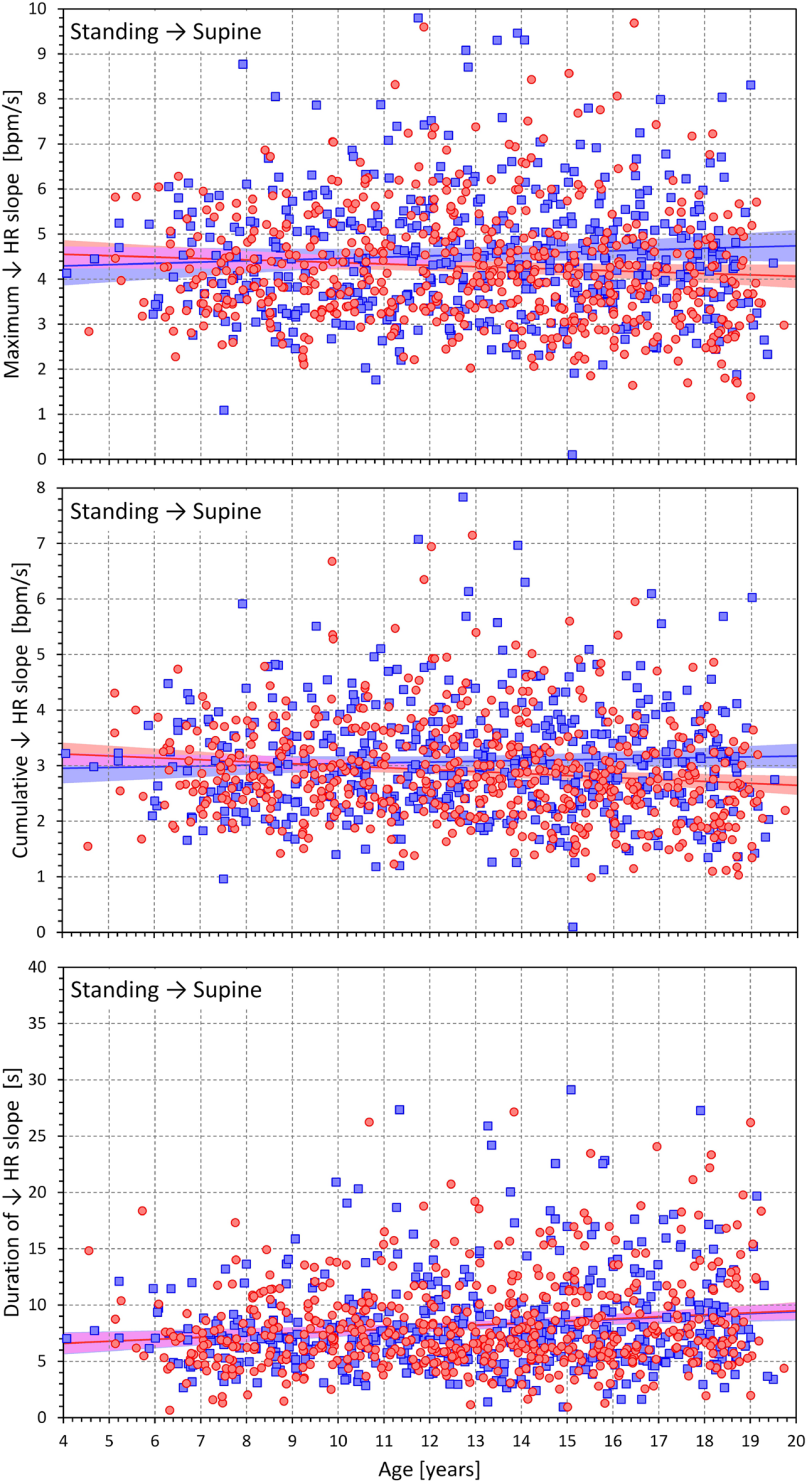
Dependency of heart rate deceleration slopes on the age of study participants. Maximum heart rate slopes (top panel), cumulative heart rate slopes (middle panel), and the duration of heart rate slopes (bottom panel) measured during the position change from standing to supine related to the age of study subjects. The layout of the figure and the meaning of the symbols, lines, and bands are the same as in Fig. 5.

The relationship between age and the heart rate changes during supine → standing transitions are shown in Fig. 5, the same relationship during the standing → supine transition is shown in Fig. 6. The figures show that there is numerically little influence of age and that the overall differences between sexes are minor compared to the inter-subject spread of the measurements.

**Figure 7:**
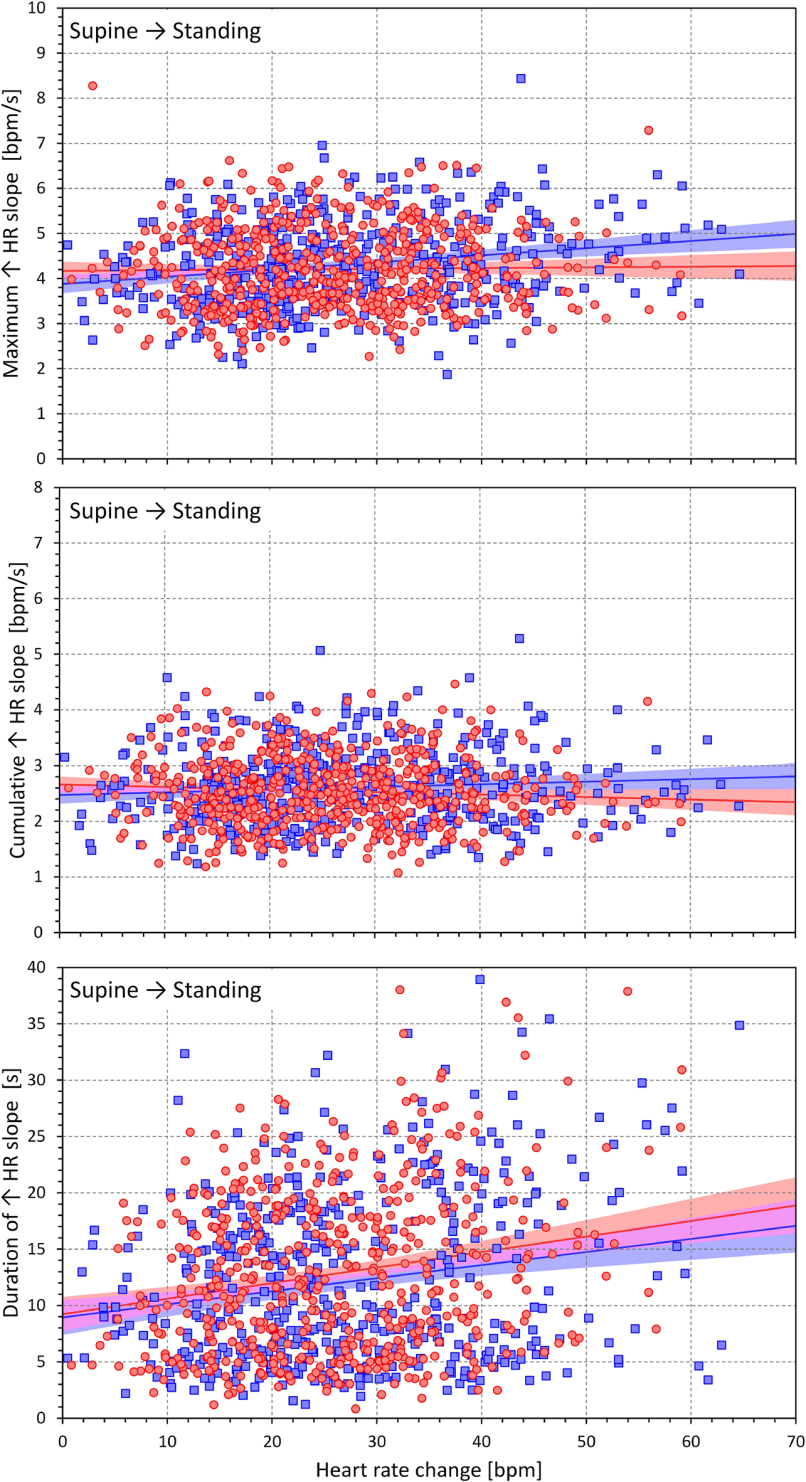
Dependency of heart rate acceleration slopes on heart rate changes. Maximum heart rate slopes (top panel), cumulative heart rate slopes (middle panel), and the duration of heart rate slopes (bottom panel) measured during the position change from supine to standing related to the heart rate changes in individual study subjects. The layout of the figure and the meaning of the symbols, lines, and bands are the same as in Fig. 5.

**Figure 8:**
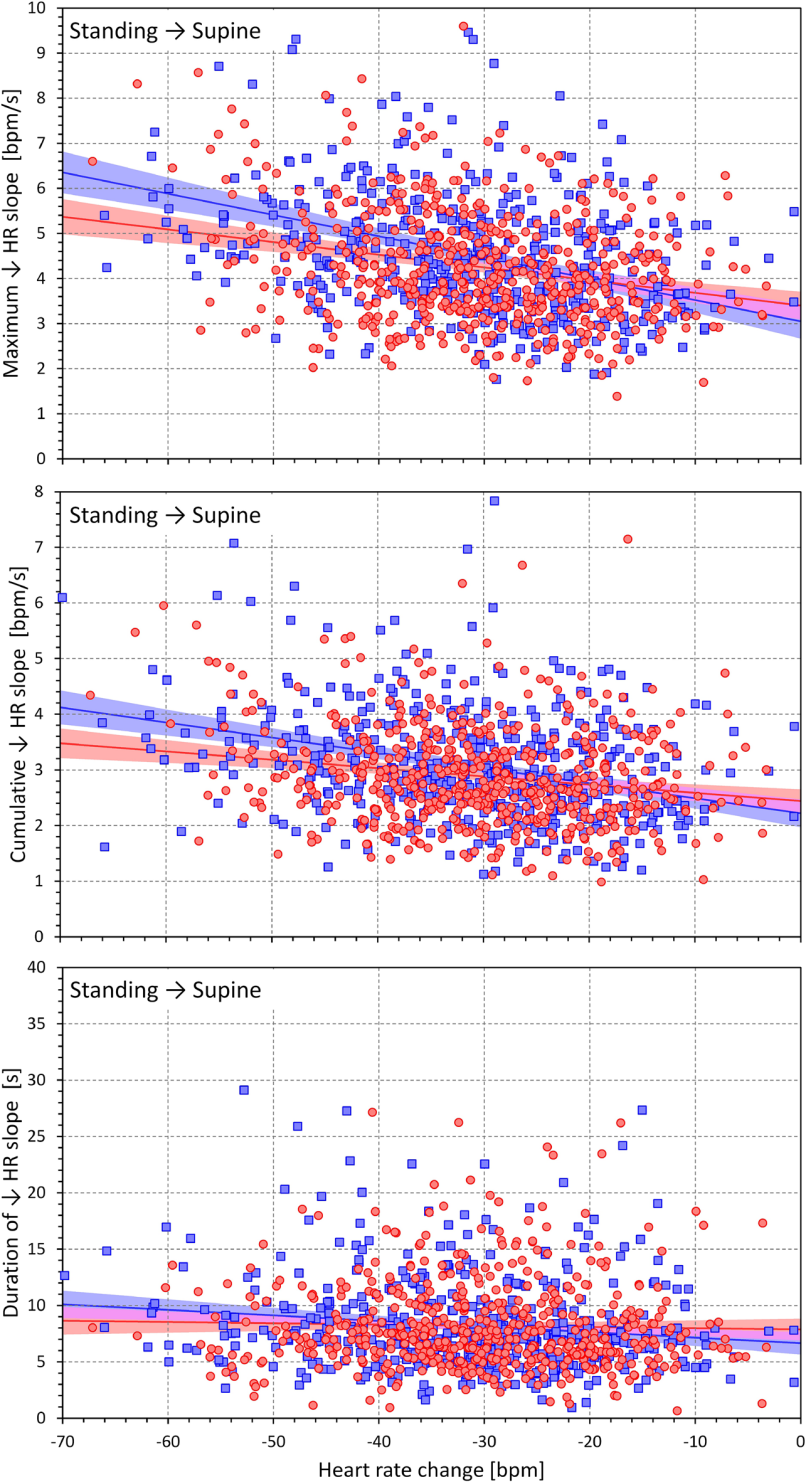
Dependency of heart rate deceleration slopes on heart rate changes. Maximum heart rate slopes (top panel), cumulative heart rate slopes (middle panel), and the duration of heart rate slopes (bottom panel) measured during the position change from standing to supine related to the heart rate changes in individual study subjects. The layout of the figure and the meaning of the symbols, lines, and bands are the same as in Fig. 5.

Figures 7 and 8 show the same heart rate change characteristics as in Figs. 5 and 6 but related to the heart rate change during postural transition rather that to the age of the subjects. Generally, the relationships were more strongly expressed. Somewhat surprisingly, the maximum and cumulative heart rate slopes were more strongly dependent on heart rate change during rate deceleration (standing → supine, Fig. 8) compared to rate acceleration (supine → standing, Fig. 7).

### Comparison of heart rate acceleration and deceleration

**Figure 9:**
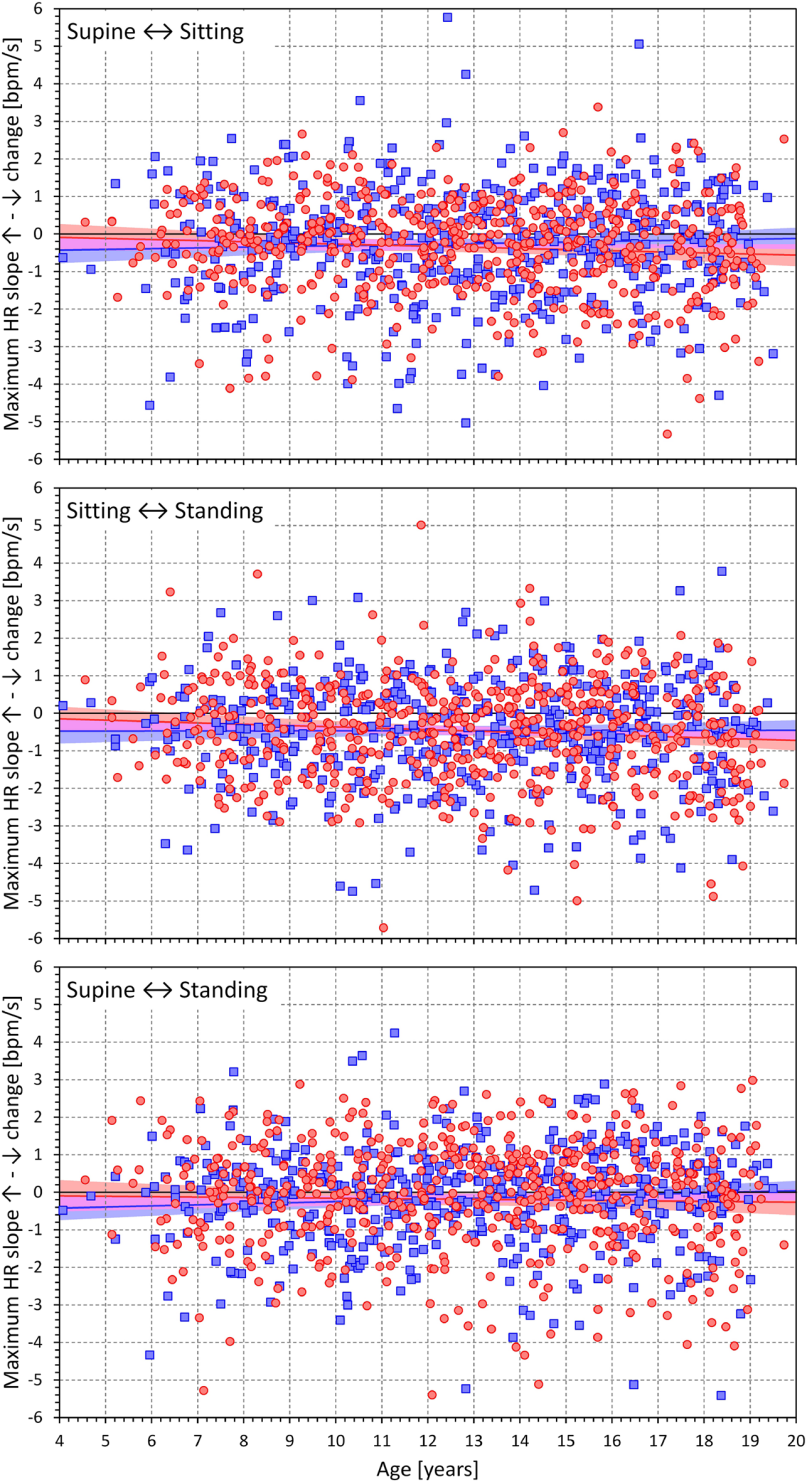
Differences between maximum heart rate acceleration and deceleration slopes. The differences between (absolute values) maximum heart rate slopes during rate acceleration and deceleration related to the age of individual subjects. The top, middle, and bottom panels show the acceleration—deceleration differences for position changes between supine and sitting, sitting and standing, and supine and standing, respectively. In each panel, the meaning of the symbols, lines, and bands is the same as in Fig. 5.

**Figure 10:**
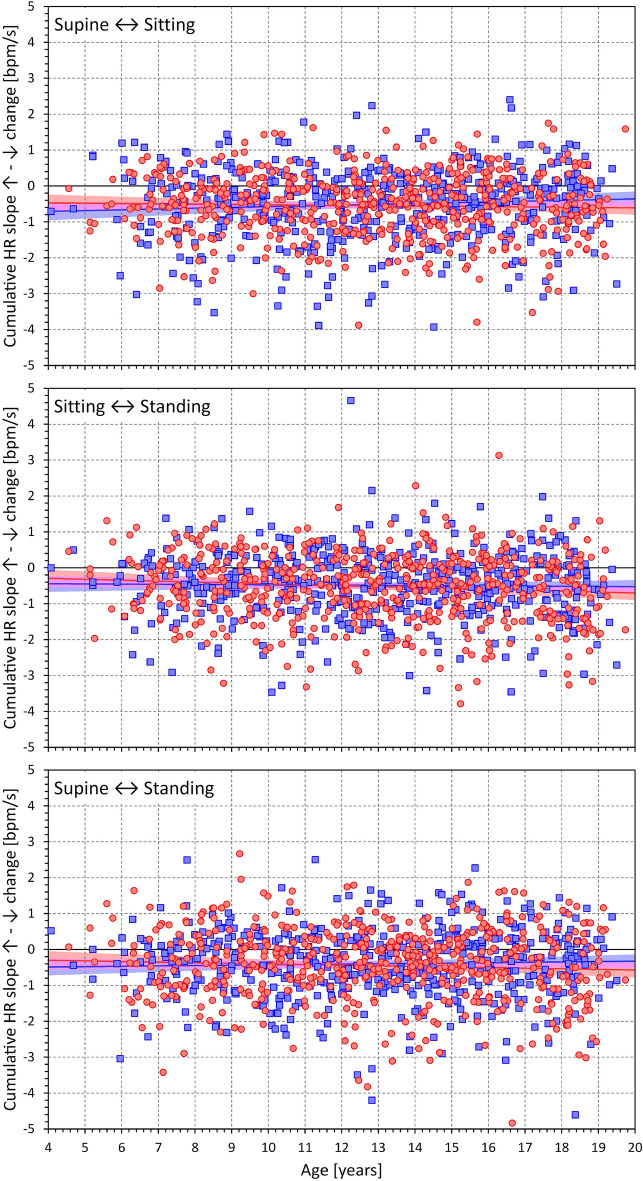
Differences between cumulative heart rate acceleration and deceleration slopes. The differences between (absolute values) cumulative heart rate slopes during rate acceleration and deceleration related to the age of individual subjects. The layout of the figure and the meaning of the symbols, lines, and bands are the same as in Fig. 9.

**Figure 11:**
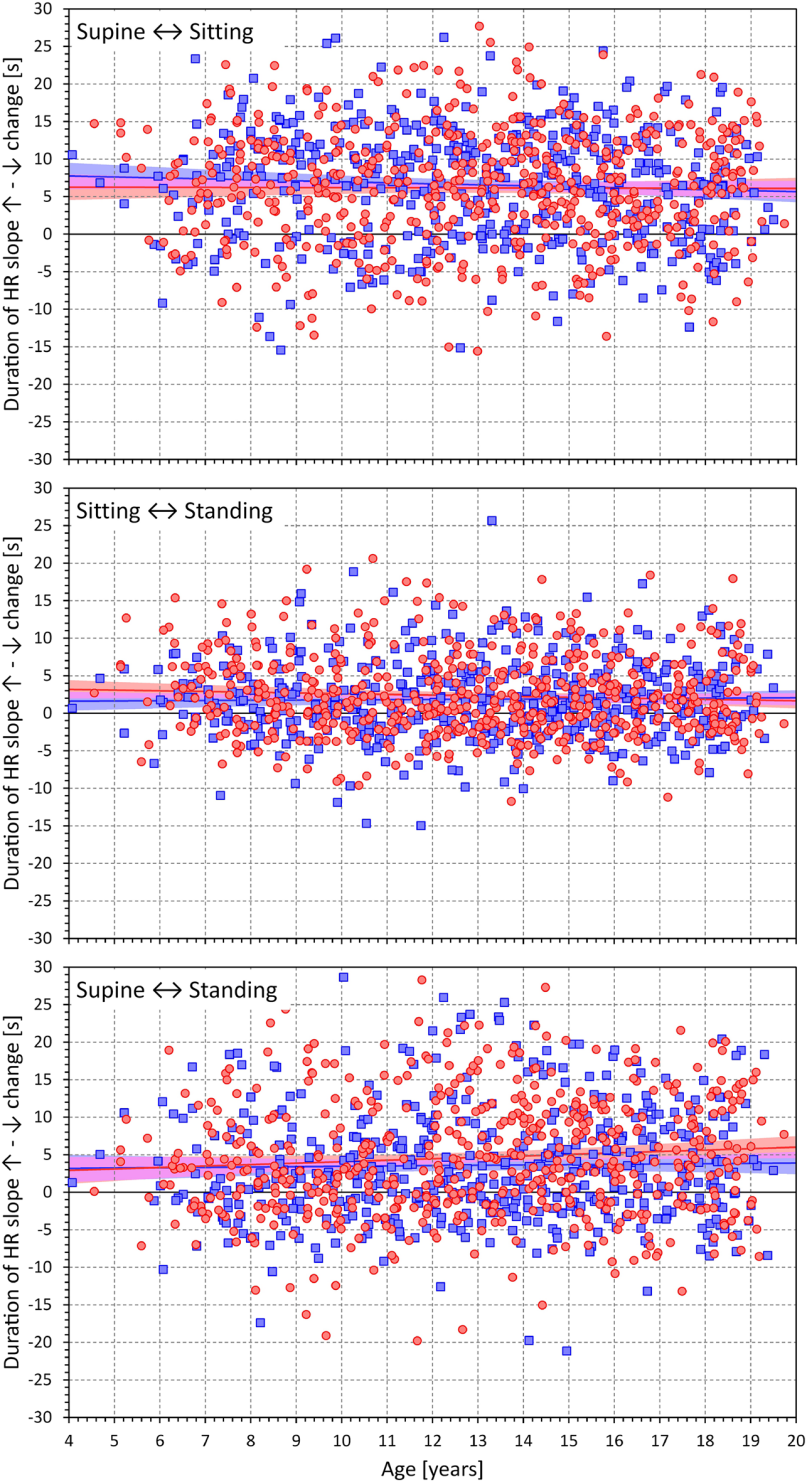
Differences between the duration of heart rate slopes during rate acceleration and deceleration. The differences between the duration of heart rate slopes during rate acceleration and deceleration related to the age of individual subjects. The layout of the figure and the meaning of the symbols, lines, and bands are the same as in Fig. 9.

Comparison of heart rate acceleration and heart rate deceleration is shown in Table 4 and Figs. 9 (position changes supine ↔ sitting, i.e., the differences between heart rate acceleration during the position change supine → sitting and heart rate deceleration during the position change sitting → supine), 10 (position changes sitting ↔ standing), and 11 (position changes supine ↔ standing). As seen in Table 4 and Figs. 9, 10 and 11, the slopes of heart rate deceleration (both maximal and cumulative) were statistically significantly steeper compared to the acceleration slopes while their durations were statistically significantly longer.

**Table 4:** Differences between heart rate acceleration and deceleration.

	Females	*P*-value	Males	*P*-value	*P*-value all	*P*-value F versus M
Supine ↔ Sitting						
Maximum slope [bpm/s]	− 0.207 ± 1.382	*0.0179*	− 0.410 ± 1.697	< *0.0001*	< *0.0001*	*0.0129*
Cumulative slope [bpm/s]	− 0.506 ± 1.040	< *0.0001*	− 0.568 ± 1.213	< *0.0001*	< *0.0001*	0.3901
Slope duration [s]	6.433 ± 7.400	< *0.0001*	6.304 ± 7.688	< *0.0001*	< *0.0001*	0.8845
Sitting ↔ Standing						
Maximum slope [bpm/s]	− 0.406 ± 1.377	< *0.0001*	− 0.526 ± 1.460	< *0.0001*	< *0.0001*	0.0871
Cumulative slope [bpm/s]	− 0.459 ± 0.942	< *0.0001*	− 0.550 ± 1.099	< *0.0001*	< *0.0001*	0.1201
Slope duration [s]	2.057 ± 5.238	< *0.0001*	2.203 ± 5.460	< *0.0001*	< *0.0001*	0.6985
Supine ↔ Standing						
Maximum slope [bpm/s]	− 0.098 ± 1.470	0.9813	− 0.253 ± 1.833	0.1330	0.3054	0.2384
Cumulative slope [bpm/s]	− 0.364 ± 0.956	< *0.0001*	− 0.487 ± 1.179	< *0.0001*	< *0.0001*	0.1425
Slope duration [s]	4.463 ± 7.706	< *0.0001*	3.806 ± 8.051	< *0.0001*	< *0.0001*	0.0879

Differences (between absolute values) of acceleration minus deceleration indices. Intra-subject differences are shown as mean ± standard deviation. *P*-values are shown for each sex as well as for both sexes combined (*p*-value all) testing the difference from 0. *P*-value F versus M shows comparison between sexes. bpm—beats per minute. Statistically significant *P*-values are shown in italics.

The intra-subject differences between acceleration and deceleration slopes and their durations were not systematically statistically significant between sexes and were not systematically significantly correlated with age. Specifically, in females, the difference in the cumulative slopes was significantly correlated with age only for the supine ↔ sitting differences, r = 0.1046 (*p* = 0.0141, CI 0.0206–0.1872), while all the differences in the duration of slopes were significantly positively correlated with age, r = 0.1094 (*p* = 0.0103, CI 0.0253–0.1919), r = 0.1619 (*p* = 0.0001, CI 0.0783–0.2433), and r = 0.1588 (*p* = 0.0002, CI 0.0752–0.2403) for the supine ↔ sitting, sitting ↔ standing, and supine ↔ standing differences, respectively. In males, only the maximum slope differences were negatively correlated with age, r =  − 0.1399 (*p* = 0.0018, CI − 0.2262 to − 0.0515) and r =  − 0.1260 (*p* = 0.0051, CI − 0.2127 to − 0.0373) for the supine ↔ sitting and sitting ↔ standing differences, respectively; with the supine ↔ sitting and sitting ↔ standing differences in the cumulative slopes also being negatively correlated with age, r =  − 0.0990 (*p* = 0.0279, CI − 0.1863 to − 0.0102) and r =  − 0.1149 (*p* = 0.0107, CI − 0.2018 to − 0.0261), respectively.

Visual judgement of Figs. 3 and 4 also shows that heart rate overshoots preceded and followed the position changes leading to heart rate deceleration and acceleration, respectively. We have not quantified these rate overshoots in the present study.

## Discussion

The study provided three surprising and unexpected observations. Firstly, contrary to our initial working hypothesis, the observed speed of heart rate changes did not increase with age. On the contrary, when observing statistical differences between age-defined tertiles, we frequently noted the speed of the heart rate changes to be numerically smaller in older subjects. The speed of the heart rate changes also showed very different overall profiles in comparison to the profiles of heart rate (see Figs. 3 and 4). Secondly, heart rate accelerations and decelerations showed unexpected asymmetry. Intuitively, because of known slower sympathetic reaction compared to vagal dynamics^1,15,16^, we expected that vagal withdrawal during heart rate acceleration will occur faster than sympathetic withdrawal and vagal charge during heart rate deceleration. The opposite was found in the measurements. Finally, we were surprised to find only negligible heart rate differences between females and males without any systematic statistically significant differences ^17,18^. The sex heart rate differences were not similar to those found in adults under similar investigative protocol of postural provocations^17^.

The observations that we made seem to suggest that the elevated heart rate in young children is not caused by insufficiently development vagal branch of the autonomic nervous system. If the vagal system was progressively developing during childhood, the very fast heart rate changes of some bpm per second would unlikely be seen in the young children. Because of the slow speed of sympathetic reactions^1^, it would be difficult to attribute such rapid rate changes to sympathetic reflexes. The vagal reflexes thus seem to differ little between younger and older children. This is also fully consistent with numerous studies that assessed the early development of psychology and of cognitive reactions by quantification of vagal reactions (e.g., by studying the extent of respiratory sinus arrhythmia)^19–22^. Links between over-reactive vagal system and sudden infant death syndrome have also been discussed^23^.

At the same time, elevated heart rate in younger children is well known and has indeed been seen in our data. At supine rest, the heart rate in the age-defined tertiles of our population decreased, on average, by some 8 bpm at each step. The differences were less marked at sitting positions and almost negligible during standing. All this, combined with the similarity of rapid vagal reflexes, suggests that compared to the parasympathetic system, the sympathetic system and mainly the interplay between the vagal and sympathetic systems develops more slowly during childhood^6^ and that even at rest, younger children are under constant sympathetic drive. Unsupported sitting and standing require activation of paravertebral muscles. Thus, similar to head-up tilt^24^ and mental stress^25^, postural changes^26^ lead to sympathetic tone increase. Near saturation of sympathetic drive might explain the similar and almost age-independent heart rate levels during standing while residual sympathetic drive at rest is likely responsible for the age-related heart rate differences during the supine position.

Partially saturated sympathetic drive seems also likely to react more slowly when responding to vagal withdrawal during postural challenges. Such a delayed response would explain the asymmetry of heart rate changes with faster heart rate deceleration compared to acceleration. Slower sympathetic charges would also lead to a prolonged time needed to reach autonomic stability after postural changes. This would explain the overshoots seen in the development of heart rate after episodes of rate acceleration (Fig. 3). Tiny rate change overshoots preceding the episodes of heart rate deceleration might be noted in Fig. 4. These overshoots were likely caused by physical efforts due to body movements during the actual postural changes. Replicating the analyses of the speed of heart rate changes in adult data might provide further insight into the acceleration/deceleration asymmetry.

The observation of negligible sex differences in heart rates is at odds with some previous publications^27^ that found, among older children, rate differences between females and males similar to the sex differences observed in adult populations^17^. Some aspects of our investigations might have contributed to the lack of such sex differences in our data. The requirements of a strictly enforced investigative protocol are not only likely to lead to some sympathetic input but might have also been perceived differently by adolescent females compared to males, similar to the sex differences in autonomic reactions to physical challenges in adults^17^. That would make our data difficult to compare with heart rate data derived from long-term recordings obtained without any prescribed and strictly enforced procedures.

Apart from the contribution to the physiologic understanding of the autonomic development in children, the methodology and results of our study might also be helpful for further assessment of paediatric vagal reactions. In addition to the so-called newborn infant parasympathetic evaluation index^28^ and other techniques based on respiratory sinus arrhythmia and related investigations of heart period variability, assessment of the speed of heart rate changes might be helpful in assessing vagal tone and autonomic responsiveness in children. The same investigations might also contribute to the diagnoses of different autonomopathies^29^ although at present, we can only propose this as an untested conjecture.

Different methods to quantify heart rate acceleration and deceleration have previously been applied to monitor disease progression in patients with a variety of neuropathic conditions^30–32^. Many of these approaches quantified heart rate changes somewhat indirectly by employing standard heart rate variability indices such as the statistics of the differences between successive RR intervals. Specific methods relying on heart rate deceleration were proposed and successfully applied to risk stratification in patients with ischaemic heart disease^33–35^. It would be interesting to apply the methods based on short-term heart rate regression slopes also in these scenarios.

### Limitations

Several limitations of the present study need to be considered. The study did not collect blood samples and we were unable to measure the levels of sex hormones. Although we collected data on secondary sex signs of the participants, we have not used these in the present study. While resting heart rate might be influenced by sex hormones, particularly in female participants^36^, the principal finding of the age-independent speed of heart rate changes is clearly not influenced by the lack of hormonal data. We have also not considered the menstrual cycle variations in female subjects^37^. Again, incorporation of such data would not change our main findings. We have not correlated the measurements with body mass index which might be valid topic of future sub-analyses^38^. Similarly, it might be interesting to consider the influence of physical and sport activity (e.g., measured by the time devoted to active sport training per week)^39^. Regrettably, limited study funding did not permit us to incorporate continuous blood pressure measurements which would allow assessing non-invasive baroreflex sensitivity^40^. For the same reason, we were able to study each subject only once and thus cannot comment on intra-subject reproducibility of the measurements which would be of importance when considering potential clinical utility of the measurements^41^. Figure 3 shows that heart rate values were not entirely reproducible in individual postural positions. This was likely caused by sequential effects of the study protocol.

## Conclusion

Plausible interpretation of the study results suggests that in children between the ages of approximately 6–19 years, vagal control of heart rate is already largely developed. In other words, our data do not support the previously made suggestions that substantial development of vagal heart rate control occurs during these age years. Further, our data offer a conclusion that the gradual decrease of resting heart rate during childhood and adolescence is caused by lower cardiac sympathetic influence. That is, based on these data, we propose that during the ages 6–19 years, vagal control of heart rate is already developed but that the interplay between both branches of the autonomic nervous system gradually develops allowing the vagal control to dominate the sympathetic control during rest. The study further observed that the speed of posture-induced heart rate deceleration appeared faster in comparison to posture-induced heart rate acceleration. The potential of clinical utility of the proposed method for measuring the speed of heart rate changes requires further elucidation.

### Supplementary Information

Supplementary Tables.

## Data Availability

The raw data supporting the conclusions of this article will be made available by the authors, without undue reservation but pending the approval by the Ethics Committee of University Hospital Brno. Requests for the raw data are to be sent to the corresponding author together with a plan for proposed analyses.
