# Supplementary material for: Speed of heart rate changes during postural provocations in children and adolescents
Source: Sci Rep. 2024 May 24;14:11938. doi: 10.1038/s41598-024-62000-7 (PMC11126691; doi:10.1038/s41598-024-62000-7)
Supplement: Supplementary file 1 — Supplementary Tables. [file 41598_2024_62000_MOESM1_ESM.pdf]

Speed of heart rate changes during postural provocations in children and adolescents

by

Martina Šišáková[1,2], Kateřina Helánová[1,2], Katerina Hnatkova[3],

Irena Andršová[1,2], Tomáš Novotný[1,2], Marek Malik[3,2]

[1] Department of Internal Medicine and Cardiology, University Hospital Brno,

Jihlavská 20, 625 00 Brno, Czech Republic

[2] Department of Internal Medicine and Cardiology, Faculty of Medicine,

Masaryk University, Jihlavská 20, 625 00 Brno, Czech Republic

[3] National Heart and Lung Institute, Imperial College,

72 Du Cane Rd, Shepherd's Bush, London W12 0NN, England

Supplementary tables

Supplementary table 1

Supine → Sitting

	Female	Male	p-value (F vs M)
Maximum slope [bpm/s]			
Age < 11 years	4.06 ± 0.88	4.06 ± 0.96	0.7090
Age 11-15 years	4.09 ± 0.89	4.06 ± 1.20	0.4160
Age > 15 years	3.81 ± 0.83	3.92 ± 0.86	0.2650
p-value (age groups)	0.0024	0.4048	
Averaged slope [bpm/s]			
Age < 11 years	2.39 ± 0.51	2.48 ± 0.74	0.5320
Age 11-15 years	2.43 ± 0.56	2.49 ± 0.82	0.8010
Age > 15 years	2.23 ± 0.53	2.39 ± 0.57	0.0070
p-value (age groups)	0.0002	0.4311	
Slope duration [s]			
Age < 11 years	13.06 ± 5.63	13.58 ± 5.26	0.5400
Age 11-15 years	14.53 ± 6.87	14.31 ± 7.03	0.8550
Age > 15 years	16.54 ± 7.74	15.48 ± 8.08	0.2330
p-value (age groups)	<0.0001	0.0261	
Rate change [bpm]			
Age < 11 years	8.87 ± 6.78	7.21 ± 6.35	0.0180
Age 11-15 years	14.65 ± 7.50	13.42 ± 8.67	0.0530
Age > 15 years	16.26 ± 8.61	18.31 ± 8.98	0.0190
p-value (age groups)	<0.0001	<0.0001	

Absolute values of heart rate change measurements shown as mean ± standard deviation. P-values “F vs M” show statistical comparison of both sexes, “age groups” show inter-sex comparisons of age-tertiles, bpm – beats per minute.

Supplementary table 2

Sitting → Standing

	Female	Male	p-value (F vs M)
	Maximum slope [bpm/s]		
Age < 11 years	3.62 ± 0.93	3.44 ± 0.82	0.0740
Age 11-15 years	3.72 ± 1.02	3.88 ± 1.46	0.4450
Age > 15 years	3.54 ± 1.09	3.59 ± 0.89	0.3110
p-value (age groups)	0.1817	0.0056	
	Averaged slope [bpm/s]		
Age < 11 years	2.38 ± 0.63	2.26 ± 0.54	0.0880
Age 11-15 years	2.37 ± 0.66	2.49 ± 1.23	0.5000
Age > 15 years	2.26 ± 0.69	2.32 ± 0.67	0.4990
p-value (age groups)	0.1201	0.1622	
	Slope duration [s]		
Age < 11 years	7.76 ± 4.01	8.47 ± 4.23	0.1140
Age 11-15 years	9.51 ± 5.27	9.87 ± 5.43	0.5410
Age > 15 years	10.41 ± 5.53	9.86 ± 5.69	0.2880
p-value (age groups)	<0.0001	0.0984	
	Rate change [bpm]		
Age < 11 years	6.49 ± 5.45	8.56 ± 5.20	0.0002
Age 11-15 years	8.99 ± 5.94	10.29 ± 6.24	0.0200
Age > 15 years	7.07 ± 7.07	8.74 ± 6.70	0.0190
p-value (age groups)	0.0003	0.0150	

Absolute values of heart rate change measurements shown as mean ± standard deviation. P-values “F vs M” show statistical comparison of both sexes, “age groups” show inter-sex comparisons of age-tertiles, bpm – beats per minute.

Supplementary table 3

Standing → Sitting

	Female	Male	p-value (F vs M)
	Maximum slope [bpm/s]		
Age < 11 years	4.14 ± 1.20	4.00 ± 1.20	0.1490
Age 11-15 years	4.14 ± 1.34	4.13 ± 1.26	0.8390
Age > 15 years	3.83 ± 1.08	4.39 ± 1.27	<0.0001
p-value (age groups)	0.0279	0.0056	
	Averaged slope [bpm/s]		
Age < 11 years	2.91 ± 0.87	2.84 ± 0.86	0.2560
Age 11-15 years	2.86 ± 0.91	2.86 ± 0.92	0.9530
Age > 15 years	2.61 ± 0.74	3.04 ± 0.87	<0.0001
p-value (age groups)	0.0018	0.0373	
	Slope duration [s]		
Age < 11 years	6.93 ± 3.26	6.53 ± 3.27	0.2480
Age 11-15 years	6.97 ± 2.69	7.73 ± 4.09	0.4970
Age > 15 years	7.63 ± 3.10	7.37 ± 3.11	0.3480
p-value (age groups)	0.0340	0.0247	
	Rate change [bpm]		
Age < 11 years	11.00 ± 5.99	11.36 ± 6.34	0.5350
Age 11-15 years	12.98 ± 6.10	15.20 ± 8.21	0.0110
Age > 15 years	11.66 ± 6.48	12.64 ± 7.21	0.0830
p-value (age groups)	0.0031	<0.0001	

Absolute values of heart rate change measurements shown as mean ± standard deviation. P-values "F vs M" show statistical comparison of both sexes, "age groups" show inter-sex comparisons of age-tertiles, bpm – beats per minute.

Supplementary table 4

Sitting → Supine

	Female	Male	p-value (F vs M)
	Maximum slope [bpm/s]		
Age < 11 years	4.35 ± 1.20	4.15 ± 1.21	0.1680
Age 11-15 years	4.33 ± 1.37	4.56 ± 1.84	0.5930
Age > 15 years	3.91 ± 1.21	4.56 ± 1.32	<0.0001
p-value (age groups)	0.0003	0.0158	
	Averaged slope [bpm/s]		
Age < 11 years	2.97 ± 0.82	2.87 ± 0.83	0.3140
Age 11-15 years	2.96 ± 0.92	3.09 ± 1.12	0.6290
Age > 15 years	2.64 ± 0.87	3.07 ± 0.95	<0.0001
p-value (age groups)	<0.0001	0.2123	
	Slope duration [s]		
Age < 11 years	7.48 ± 2.86	7.27 ± 2.94	0.2510
Age 11-15 years	7.95 ± 3.96	7.96 ± 3.39	0.4390
Age > 15 years	9.45 ± 5.15	9.23 ± 4.94	0.7320
p-value (age groups)	0.0022	0.0017	
	Rate change [bpm]		
Age < 11 years	12.44 ± 7.17	11.14 ± 5.90	0.1150
Age 11-15 years	20.48 ± 8.58	17.51 ± 8.60	0.0006
Age > 15 years	24.38 ± 8.93	23.99 ± 10.15	0.5120
p-value (age groups)	<0.0001	<0.0001	

Absolute values of heart rate change measurements shown as mean ± standard deviation. P-values “F vs M” show statistical comparison of both sexes, “age groups” show inter-sex comparisons of age-tertiles, bpm – beats per minute.
